# Comparative Analysis of Secretome Profiles of Manganese(II)-Oxidizing Ascomycete Fungi

**DOI:** 10.1371/journal.pone.0157844

**Published:** 2016-07-19

**Authors:** Carolyn A. Zeiner, Samuel O. Purvine, Erika M. Zink, Ljiljana Paša-Tolić, Dominique L. Chaput, Sajeet Haridas, Si Wu, Kurt LaButti, Igor V. Grigoriev, Bernard Henrissat, Cara M. Santelli, Colleen M. Hansel

**Affiliations:** 1 School of Engineering and Applied Sciences, Harvard University, Cambridge, Massachusetts, United States of America; 2 Environmental Molecular Sciences Laboratory, Pacific Northwest National Laboratory, Richland, Washington, United States of America; 3 Biological Sciences Laboratory, Pacific Northwest National Laboratory, Richland, Washington, United States of America; 4 Department of Mineral Sciences, National Museum of Natural History, Smithsonian Institution, Washington, DC, United States of America; 5 U.S. Department of Energy Joint Genome Institute, Walnut Creek, California, United States of America; 6 Architecture et Fonction des Macromolécules Biologiques, UMR7257, Centre National de la Recherche Scientifique and Aix-Marseille Université, 13288 Marseille Cedex 9, France; 7 Department of Biological Sciences, King Abdulaziz University, P.O. Box 80203, Jeddah, 21589, Saudi Arabia; 8 Department of Earth Sciences, University of Minnesota, Minneapolis, Minnesota, United States of America; 9 Department of Marine Chemistry and Geochemistry, Woods Hole Oceanographic Institution, Woods Hole, Massachusetts, United States of America; Georg-August-University of Göttingen Institute of Microbiology & Genetics, GERMANY

## Abstract

Fungal secretomes contain a wide range of hydrolytic and oxidative enzymes, including cellulases, hemicellulases, pectinases, and lignin-degrading accessory enzymes, that synergistically drive litter decomposition in the environment. While secretome studies of model organisms such as *Phanerochaete chrysosporium* and *Aspergillus* species have greatly expanded our knowledge of these enzymes, few have extended secretome characterization to environmental isolates or conducted side-by-side comparisons of diverse species. Thus, the mechanisms of carbon degradation by many ubiquitous soil fungi remain poorly understood. Here we use a combination of LC-MS/MS, genomic, and bioinformatic analyses to characterize and compare the protein composition of the secretomes of four recently isolated, cosmopolitan, Mn(II)-oxidizing Ascomycetes (*Alternaria alternata* SRC1lrK2f, *Stagonospora* sp. SRC1lsM3a, *Pyrenochaeta* sp. DS3sAY3a, and *Paraconiothyrium sporulosum* AP3s5-JAC2a). We demonstrate that the organisms produce a rich yet functionally similar suite of extracellular enzymes, with species-specific differences in secretome composition arising from unique amino acid sequences rather than overall protein function. Furthermore, we identify not only a wide range of carbohydrate-active enzymes that can directly oxidize recalcitrant carbon, but also an impressive suite of redox-active accessory enzymes that suggests a role for Fenton-based hydroxyl radical formation in indirect, non-specific lignocellulose attack. Our findings highlight the diverse oxidative capacity of these environmental isolates and enhance our understanding of the role of filamentous Ascomycetes in carbon turnover in the environment.

## Introduction

Fungal secretomes are reservoirs of a diverse suite of extracellular enzymes and reactive metabolites that are specialized to breakdown recalcitrant plant and animal material in the environment. In particular, fungi secrete a wide range of hydrolytic and oxidative enzymes, including cellulases, hemicellulases, pectinases, and lignin-degrading accessory enzymes that generate reactive oxygen species (ROS), which synergistically drive litter decomposition in natural systems and can be harnessed for industrial applications [1–4]. As such, fungal secretomes are critical drivers of global carbon cycling and climate dynamics, as well as essential mediators in renewable energy production.

The continued advancement of analytical techniques in microbial genomics, transcriptomics, proteomics, and metabolomics has allowed for deeper interrogation of the mechanistic underpinnings of complex microbially-mediated processes within the environment, generating a considerable amount of data and facilitating new insights into microbial metabolism. In particular, comparative proteomics has proven to be a valuable tool in investigating the response of fungal secretomes to different growth conditions and environmental stimuli, including substrate composition [5–8], growth phase [9], lifestyle [10], starvation [11], and response of a fungal pathogen to its host [12] or vice versa [13]. These comprehensive secretome characterizations have included both Basidiomycetes and Ascomycetes, primarily focusing on elucidating the mechanisms of demonstrated lignin degradation capacity of white-rot Basidiomycetes [8, 14, 15] and optimizing the production of cellulose-degrading enzymes in model Ascomycete fungi in the *Aspergillus* [9, 11] and *Fusarium* [12] genera.

While our knowledge surrounding the hydrolytic and oxidative enzymes secreted by these organisms is expanding rapidly, few studies have extended secretome characterization efforts beyond model organisms to environmental isolates, and as such, the mechanisms underlying their contribution to recalcitrant carbon degradation in terrestrial systems remain poorly understood. In addition, few studies have directly compared the secretome composition of multiple organisms side-by-side (see [16] for an example using yeasts and [8] for wood decay Basidiomycetes), a valuable tool in investigating the diversity in extracellular hydrolytic and oxidative processes among co-occurring fungi in natural lignocellulose-degrading communities.

In this study, we begin to address these knowledge gaps by investigating the protein composition of the secretomes of four cosmopolitan, Mn(II)-oxidizing, filamentous Ascomycete fungi that we have recently isolated from various terrestrial environments. Mn(II)-oxidizing fungi are of engineering interest due to their ability to aid in the bioremediation of metal-contaminated waters [17, 18]. Three of the organisms, *Alternaria alternata* SRC1lrK2f, *Stagonospora* sp. SRC1lsM3a, and *Pyrenochaeta* sp. DS3sAY3a, were isolated from passive coal mine drainage treatment systems in central Pennsylvania, USA, in which microbial Mn oxide formation is actively used to remove toxic metals from contaminated drainage waters via adsorption and settling [17]. The fourth species, *Paraconiothyrium sporulosum* AP3s5-JAC2a, was isolated from a freshwater lake in Massachusetts, USA, that was historically contaminated with high concentrations of metals, including iron and manganese, and nutrients [19].

Mn(II)-oxidizing fungi are also of commercial and industrial interest due to their potential to utilize the oxidation of Mn(II) in the breakdown of recalcitrant lignocellulosic plant material [3, 4]. For instance, white-rot Basidiomycetes such as *Phanerochaete chyrsosporium* directly couple Mn(II) oxidation to lignocellulose oxidation, and this process is dictated by extracellular enzymes and ROS in the secretome [20–23]. While the Ascomycetes investigated in this study have demonstrated cellulose degradation capacity (C.M. Santelli, unpublished data), the mechanisms by which they catalyze this process remain unknown. Furthermore, it is unclear whether these organisms’ ability to oxidize Mn(II) is linked to their ability to break down cellulose, as it is in model white-rot Basidiomycete fungi.

In addition to their engineering and industrial potential, the four Ascomycetes investigated in this study represent species with varied lifestyles that are present in soil ecosystems worldwide. *Alternaria alternata* is one of the most common species of fungi identified in soils from diverse environments across the globe and is a frequent early colonizer of plant litter [24]. It has been implicated as both a plant pathogen in food crops and an opportunistic pathogen in humans [25, 26], in addition to living a saprotrophic lifestyle on dead and decaying material [24]. *Paraconiothyrium sporulosum* also has a cosmopolitan distribution in soil [27], and coniothyrium-like fungi (including species in the genus *Paraconiothyrium*) have been identified as plant pathogens and biological control agents [28, 29]. Furthermore, *P*. *sporulosum* can promote wood degradation [30, 31]. Although species-level determination for the *Stagonospora* and *Pyrenochaeta* isolates investigated in this study has not yet proven successful, much is known about their respective genera. Fungi in the genus *Stagonospora* are widely known as aggressive pathogens of wheat (e.g., *S*. *nodorum*) [32], and others have been implicated in the degradation of aromatic compounds and lignin derivatives (e.g., *S*. *gigaspora*) [33]. Fungi in the genus *Pyrenochaeta* inhabit soil and plant debris worldwide and are well-known as pathogens of plants and occasionally humans [34, 35].

Thus, the fungi investigated herein represent non-model organisms that are ubiquitous in the environment and have the ability to degrade cellulose and generate reactive compounds such as Mn(III) aqueous complexes and solid-phase Mn(IV) oxides, which could contribute to lignin degradation. Yet, little is known about their contribution to carbon cycling in natural soils or the mechanisms and associated enzymes responsible for these degradation processes.

Here we fully characterized the protein composition of the secretomes of these four environmental isolates. Using LC-MS/MS-based comparative proteomics combined with genomic and bioinformatic analyses, we directly compared the composition and functional diversity of the secretomes among organisms, demonstrating that the fungi produce a rich yet functionally similar suite of extracellular enzymes, despite the identification of many species-specific proteins identified under the experimental conditions. Furthermore, we identified numerous lignocellulose-degrading enzymes in each of the 4 fungi that may be used as targets for future mechanistic investigations of the cellulose-degrading capacity of these organisms. This work highlights the rich functional diversity and oxidative capacity of these fungal secretomes, demonstrates the utility of comparative proteomics in interrogating diverse species, and enhances our understanding of the role of filamentous Ascomycetes in plant material turnover in the environment.

## Materials and Methods

### Fungal species and culture medium

We investigated four filamentous Ascomycete fungi that were isolated from two types of environments. Three species were isolated from passive coal mine drainage treatment systems in central Pennsylvania that attenuate high concentrations of Mn [17]: *Alternaria alternata* SRC1lrK2f, *Stagonospora* sp. SRC1lsM3a, and *Pyrenochaeta* sp. DS3sAY3a. Permission for sampling at these field sites was provided by Cliff Denholm of Stream Restoration Incorporated, a non-profit organization located in Mars, PA. The fourth species was isolated from Ashumet Pond, Massachusetts, a natural freshwater lake [19]: *Paraconiothyrium sporulosum* AP3s5-JAC2a. This field site was historically polluted with elevated concentrations of phosphate and metals, including Fe and Mn, and is currently undergoing remediation. No permission was required for field sampling at this site; samples were taken on public lands adjacent to the State Boat Landing. None of the field sampling in this study involved endangered or protected species.

All four fungi are classified in the *Pezizomycotina* sub-phylum. We believe the following classifications are accurate as of this writing: *A*. *alternata* belongs to the suborder *Pleosporineae* and family *Pleosporaceae* [36]; *Pyrenochaeta* sp. also belongs to the suborder *Pleosporineae* but resides within the family *Cucurbitariaceae* [36, 37]; *P*. *sporulosum* belongs to the suborder *Massarineae* and family *Montagnulaceae* [36]; and *Stagonospora* sp. similarly belongs to the suborder *Massarineae* but family *Massarinaceae* [17, 36].

All fungal species were grown in HEPES-buffered (20 mM, pH 7) AY medium, which consists of 0.25 g L^-1^ sodium acetate, 0.15 g L^-1^ yeast extract, and 1 mL L^-1^ trace element stock (10 mg L^-1^ CuSO_4_•5H_2_O, 44 mg L^-1^ ZnSO_4_•7H_2_O, 20 mg L^-1^ CoCl_2_•6H_2_O, and 13 mg L^-1^ Na_2_MoO_4_•2H_2_O) supplemented with MnCl_2_ (0–200 μM). All chemicals were reagent grade or higher. Fungal cultures were maintained on petri dishes containing agar-solidified (2% agar) AY medium with 200 μM Mn(II) (hereafter AY + Mn).

### Culture conditions and secretome harvesting

Homogenized inocula were used for all culture experiments. Inocula were prepared by aseptically removing the entire contents of a 90 mm petri dish (including fungal mycelia and associated agar) that had incubated at room temperature (20°C) until the mycelia had reached the edge of the agar. The contents were then placed in an autoclaved kitchen blender (Oster model BVLB07) with 100 mL of AY + Mn medium and homogenized on high speed for 2 minutes. On the same day that the homogenized inocula were prepared, 100 μL of the inoculum was used to inoculate 100 mL liquid cultures in AY + Mn medium.

For characterization of secretome samples, liquid cultures of each of the 4 fungi were incubated at room temperature and ambient light, without agitation, for 7, 14, or 21 days. For each fungus and each time point, individual 100 mL cultures were combined into 500 mL samples. All 500 mL samples were prepared in duplicate. Upon harvesting, bulk biomass was removed with a sterile wooden stick and discarded, and the spent medium was filtered through a 0.45 μm polyethersulfone membrane (VWR) to remove remaining cells and Mn oxides. Samples were then concentrated using a centrifugal filter with a 10 kDa, low protein adhesion membrane (EMD Millipore). Centrifugation proceeded at 2200 × g on a Sorvall RT 6000B centrifuge with H1000B swing-bucket rotor until all liquid had passed through the membrane. The resulting secretome samples were rinsed with 20 mM HEPES, pH 7 and stored at -80°C until analysis.

Protein in secretome samples was quantified using a Pierce^TM^ BCA protein assay kit (Thermo Fisher Scientific) as conducted previously [38]. The quantity of protein recovered from 500 mL secretome samples generally ranged between 200 and 1000 μg, depending on species and secretome age.

### Proteomics

#### Sample preparation

Secretome samples were prepared for LC-MS/MS proteomic analysis using a trypsin digestion protocol similar to previously described [39]. In summary, the proteins were denatured with urea (8 M) and reduced with 5 mM dithiothreitol (DTT, Sigma–Aldrich) for 30 min at 60°C. Protein alkylation was not performed in an effort to avoid negatively impacting quantitation of low abundance (e.g., secreted) proteins. The samples were then diluted 10-fold with 100 mM ammonium bicarbonate with 1 mM CaCl_2_ and then digested for 3 h at 37°C using porcine sequencing-grade trypsin (Promega) at a substrate/enzyme mass ratio of 50:1. The digestion was quenched by adding 10% trifluoroacetic acid to a final concentration of 0.1% before desalting with a C-18 solid phase extraction column (Supelco), performed using a Gilson GX-274 Liquid Handler. The resulting peptide solution was concentrated to 50 μL in a vacuum concentrator, and a BCA assay was performed to estimate the protein concentration, as above. To enable quantitation, the samples were labeled with Isobaric Tags for Relative and Absolute Quantitation (iTRAQ) 8-plex Reagent following the manufacturer’s instructions (ABSciex, PN#4352135). Quantitative aspects of this study will be presented in a separate publication. The iTRAQ labeled samples were desalted with a C-18 solid phase extraction column and then fractionated with a reverse-phase C18 column and pooled using concatenation [40]. The resulting fractions were diluted to 0.07 μg/μL and were then analyzed using LC-MS/MS.

#### LC-MS/MS analysis

The iTRAQ labeled fractions were processed on a Waters nano-Acquity dual pumping UPLC system with a custom on-line trapping system for a 5 μL injection processed at 3 μL min^-1^. The trapped sample was then reversely eluted onto the analytical column at a 300 nL min^-1^ flow rate. Both the trapping column (150 μm i.d. x 4 cm long) and analytical column (75 μm i.d. x 70 cm long) were packed in-house using Jupiter C18 media (Phenomenex) particles (5 μm for the trapping column and 3 μm for the analytical column) into 360 μm o.d. fused silica (Polymicro Technologies Inc.) with 1 cm sol-gel frits for media retention [41]. The gradient began with mobile phase A (0.1% formic acid in water) and switched to mobile phase B (0.1% formic acid in acetonitrile) with the following gradient profile (min, %B): 0, 1; 2, 8; 20, 12; 75, 30; 97, 45; 100, 95; 110, 95; 115, 1; 150, 1.

LC-MS/MS data were acquired using a LTQ Orbitrap Velos mass spectrometer (Thermo Fisher Scientific) with a custom nano-ESI interface. The ESI emitters were custom-made by chemically etching fused silica (150 μm o.d. x 20 μm i.d) [42]. The heated capillary temperature was set to 350°C with a spray voltage of 2.2 kV. Data were acquired for a total of 100 min after a 15 min delay from sample injection. Full MS spectra were acquired for 300<*m/z*<1800 at a resolution of 30K. Higher-energy collisional dissociation (HCD) MS/MS with a normalized collision energy setting of 45% (a resolution of 7.5K) was performed in the data-dependent mode using the ten most abundant parent ions, excluding single charge states.

#### Data analysis

Tandem mass spectra were deconvoluted into ASCII text files using the DeconMSn tool [43]. Spectra from all 4 fungi were then searched as a combined dataset with MSGF+ [44] using a customized protein database containing translated protein sequences from the genomes of all 4 fungal species. The protein database was amended with common contaminants (e.g., trypsin and human keratin sequences). The peptide identifications were filtered to 1% FDR (using MGSF+’s reported Q-value ≤0.01, which is derived using the standard decoy approach) [45]. Because this 1% FDR criterion carefully controls for false discovery events, we chose the minimum number of peptides for unambiguous protein identification to be 1. Identified proteins based on 1 peptide comprised 24–29% of the total number of proteins identified in the secretomes of each species in this study, but they represented merely 3–5% of the total number of identified carbon-degrading enzymes (i.e., carbohydrate-active enzymes, peptidases, and lipases) per species. Thus, 95–97% of carbon-degrading enzymes in each species were identified with 2 or more peptides.

For the database search, the iTRAQ 8-plex reporter ion conjugate mass (+304.205353 Da on Lysine residues and N-termini) was set as the static modification, and the oxidation on methionine was set as the dynamic modification. Other settings included parent mass tolerance of +/- 20 ppm, partially tryptic cleavages included, +/-1 Da parent corrections (to account for incorrect monoisotopic mass determination), MS level data centroiding, and decoy search mode enabled.

Because peptide sequences from all 4 fungi were searched against all 4 genomes simultaneously, multiple protein matches for some peptides were identified among the 4 genomes. For biological interpretation of the data, only the first protein reference identified for each peptide was used, and the function of this protein was manually checked against other protein matches for that peptide to ensure that all protein functions for a particular peptide were similar.

Functional information was derived by searching a combined database of NCBI and UniProt fungal protein sequences for proteins identified in this study using BLAST analysis. For proteins for which the highest-scoring (lowest E-value) match was hypothetical or uncharacterized, the highest-scoring non-hypothetical match was reported. Proteins with no matches (hypothetical or otherwise) having an E-value below 10^−9^ (for proteins with 1 identified peptide) or 10^−6^ (for proteins with 2+ identified peptides) were reported as hypothetical. Functional information obtained via BLAST analysis was manually checked against automatically assigned annotations in the genomes (sequenced by the Joint Genome Institute (JGI)). To facilitate this, proteins identified in this study were mapped to JGI protein IDs, which were then used to retrieve and check genome annotations. As automatically-assigned annotations often contain errors [46], emphasis was placed on functional information from the BLAST analysis, as has been done by others [8]. All proteins were then functionally categorized according to the Carbohydrate-Active Enzymes Database (CAZy) (www.cazy.org) [47] and MEROPS Peptidase Database (http://merops.sanger.ac.uk/) [48] classification systems, using UniProt family and domain designations to aid in categorization.

### Genomic analysis

#### Genome-based predicted secretomes

The genomes of each of the 4 fungal species were sequenced by the U.S. Department of Energy JGI using the Illumina platform, assembled using Allpaths-LG [49], and annotated using the JGI annotation pipeline [50]. For each species, the predicted secretome was determined based on the methods described by Emanuelsson et al. [51]. The predicted secretome as defined here is composed of proteins that contain a signal peptide as determined by SignalP 4.1 [52], have a signal peptide that indicates extracellular secretion as opposed to a mitochondrial target as determined by TargetP 1.1 [53], and have no transmembrane domains as determined by TMHMM 2.0 [54]. However, transmembrane domains were allowed when present in an extracellular signal peptide.

Functional information for predicted secretome proteins was derived as described above, using an E value of 10^−6^ when evaluating BLAST analysis results. Proteins were also categorized into CAZy and MEROPS functional groups as described above.

#### Proteins unique to each genome

We used OrthoMCL v.2.0.9 [55] with mcl inflation factor 2.0 to identify orthologous gene clusters in the 4 fungal genomes. The OrthoMCL tool constructs orthologous groups of proteins across multiple eukaryotic species using a Markov Cluster algorithm to group orthologs and paralogs. Proteins not grouped into any orthologous group were identified as singletons and designated as unique to their respective genomes. When an orthologous group was identified in which all proteins were derived the same species, we considered this to be a paralogous group, and all proteins in that group were designated as unique to that species.

### Fungal genome sequence availability

The genome sequences and annotations for the 4 fungi in this study are available at the JGI fungal genomics resource MycoCosm [56] at http://genome.jgi.doe.gov/programs/fungi/index.jsf; genomes are identified in MycoCosm as Altal1 (*A*. *alternata*), Parsp1 (*P*. *sporulosum*), Pyrsp1 (*Pyrenochaeta* sp.), and Stasp1 (*Stagonospora* sp.). Genome assemblies and annotations were also deposited at DDBJ/ENA/GenBank under the following accession numbers: LXPP00000000 (*A*. *alternata*), LXPO00000000 (*P*. *sporulosum*), LXSZ00000000 (*Pyrenochaeta* sp.), and LXTA00000000 (*Stagonospora* sp.). The versions described in this paper are LXPP01000000, LXPO01000000, LXSZ01000000, and LXTA01000000, respectively.

## Results

A complete list of the proteins experimentally identified in each of the four fungal secretomes, including assigned functions and CAZy and MEROPS classifications (for carbohydrate-active enzymes and peptidases, respectively), is presented in S1 Table. Complete lists of proteins in the genome-based predicted secretomes of each fungus are presented in S2–S5 Tables.

For the purposes of this manuscript, experimental secretome composition was evaluated for each fungus across the entire 21-day study. In other words, proteins were included if they were identified in secretomes harvested at any of the 3 time points (7, 14, or 21 days) to allow for comparison of the full suite of secretome proteins. In general, the diversity of proteins in each fungal secretome varied less than 6% across each time point and no more than 10% in any one protein functional group over the 21-day study. Thus, the suite of proteins identified in each secretome was fairly consistent over time. A detailed analysis of the temporal changes in the abundance of these proteins will be presented in a separate publication.

### Secretome size

**Fig 1:**
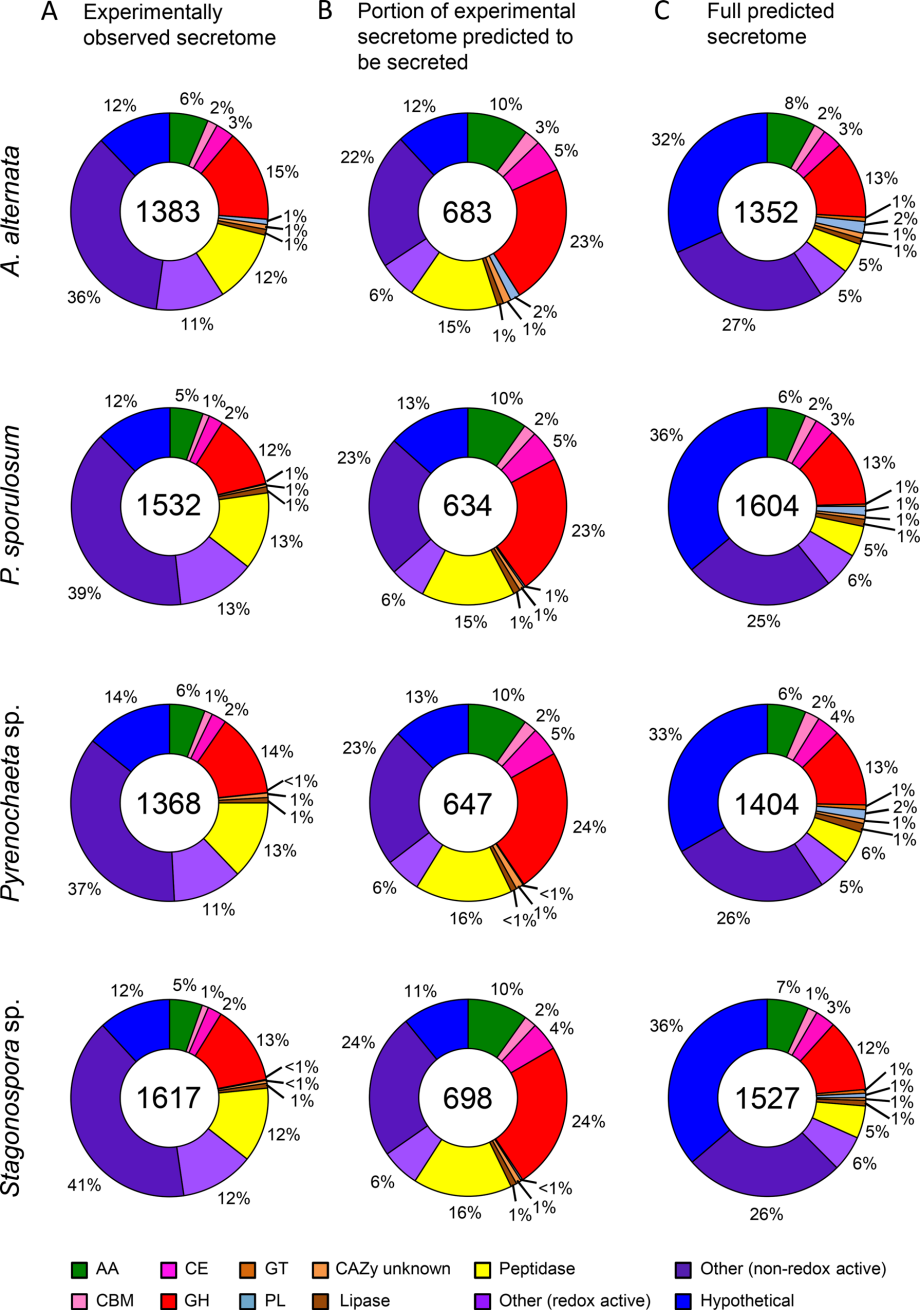
Distribution of proteins identified in secretomes of four Ascomycete fungi among broad functional groups. (A) Experimentally observed secretome. Proteins identified via LC-MS/MS over a 21-day study. (B) Portion of experimental secretome predicted to be secreted based on genome analysis (see text for further explanation). (C) Full predicted secretome based on genomes only. Total number of proteins identified for each fungus is indicated in center of circles. Abbreviations from CAZy database: AA = auxiliary activities; CBM = carbohydrate-binding module; CE = carbohydrate esterase; GH = glycoside hydrolase; GT = glucosyltransferase; PL = polysaccharide lyase.

Over 1,300 proteins were identified in the experimental secretome of each of the 4 fungi, ranging from 1,368 identified proteins in the *Pyrenochaeta* sp. secretome to 1,617 identified proteins in the *Stagonospora* sp. secretome (Fig 1A). Within CAZy and MEROPS classifications, between 518 (in the *Pyrenochaeta* sp. secretome) and 576 (in the *Stagonospora* sp. secretome) enzymes were identified. When the experimental secretomes were filtered to only include those identified proteins that are predicted to be secreted based on genomic analysis, the secretome size was reduced to <700 identified proteins for each species (Fig 1B), including 365 (in the *P*. *sporulosum* secretome) to 412 (in the *Stagonospora* sp. secretome) identified proteins that were classified within CAZy and MEROPS functional groups. The size of the full predicted secretomes (i.e., based on genomic data only) of each fungus was comparable to that of the experimental secretomes, ranging from 1,352 proteins in the *A*. *alternata* predicted secretome to 1,604 proteins in the *P*. *sporulosum* predicted secretome (Fig 1C). Within the predicted secretomes, 478 (in *A*. *alternata*) to 535 (in *P*. *sporulosum*) proteins were classified within CAZy and MEROPS functional groups.

### Secretome diversity across broad functional groups

Categorizing the proteins in each experimental secretome based on broad functional groups according to the CAZy and MEROPS databases reveals a striking similarity in secretome functional diversity among the four organisms (Fig 1A). The total proportion of CAZymes identified in the secretomes ranged from 22% in *P*. *sporulosum* to 28% in *A*. *alternata* and consisted predominantly of glycoside hydrolases (GHs; 12–15%) and redox-active auxiliary activities (AAs; 5–6%) in all four fungi. Peptidases comprised 12–13% of identified proteins in each of the fungal secretomes, while lipases comprised only 1%. Identified proteins that are not considered by the CAZy or MERPOS databases were categorized as “other” and constituted a large portion of the secretome for each fungus, ranging from 47% in *A*. *alternata* to 53% in *Stagonospora sp*. Approximately one-third of “other” identified proteins were redox-active and included dehydrogenases, oxidases, reductases, and FAD-binding proteins, among others. Non-redox active “other” proteins varied widely in predicted function and included many proteins likely of intracellular origin. Hypothetical proteins, for which no function could be predicted based on genome annotations and a BLAST analysis against sequenced fungal genomes in NCBI and UniProt, comprised 12–14% of the fungal secretomes.

When only considering experimentally identified proteins that are predicted to be secreted, the proportion of “other” proteins decreased substantially and represented only 22–24% of the secretomes, down from an average of 50% when all experimentally identified proteins were considered (Fig 1B). In the full predicted secretomes, the proportion of hypothetical proteins increased almost 3-fold, from 12–14% in the experimental secretomes up to 32–36% in the predicted secretomes (Fig 1C). In addition, the ratio of peptidases to GHs progressively decreased from an average of 0.9 in the experimental secretomes across the 4 fungi (e.g., 13%/14% in *Pyrenochaeta* sp.), to 0.7 in the portion of experimental secretomes predicted to be secreted, and finally to 0.4 in the full predicted secretomes (Fig 1). All of these observations were consistent across all 4 organisms.

Lignocellulose degrading enzymes (i.e., CAZymes) were identified in high numbers and with rich diversity in all four fungi (Table 1), representing all major CAZy classes. In the experimentally observed secretomes, the quantity and diversity of CAZymes was highest among GHs, with over 40 families represented and an average of 200 proteins identified in this class in each fungus. Redox-active AAs and carbohydrate esterases (CEs) were also well represented. In all CAZy classes except glucosyltransferases (GTs; which were poorly represented in all four organisms), the secretome of *A*. *alternata* exhibited the greatest number of identified CAZymes and the richest family diversity.

**Table 1:** Summary of identified CAZymes in secretomes of four Ascomycete fungi.

Species	CAZyme Class											
	Glycoside hydrolases (GH)		Glycosyltransferases (GT)		Carbohydrate binding modules (CBM)		Carbohydrate esterases (CE)		Polysaccharide lyases (PL)		Auxiliary activities (AA)	
	Families	Proteins	Families	Proteins	Families	Proteins	Families	Proteins	Families	Proteins	Families	Proteins
**Experimentally observed secretome**												
*A*. *alternata*	45	210	0	0	8	23	6	42	4	12	6	88
*P*. *sporulosum*	43	189	0	0	6	17	5	36	2	2	6	83
*Pyrenochaeta* sp.	40	188	0	0	7	18	6	33	1	1	5	80
*Stagonospora* sp.	41	214	0	0	6	17	5	37	1	2	6	87
**Portion of experimental secretome predicted to be secreted**												
*A*. *alternata*	41	158	0	0	6	18	6	36	4	11	6	69
*P*. *sporulosum*	39	146	0	0	4	13	5	33	2	2	5	62
*Pyrenochaeta* sp.	35	154	0	0	5	14	6	31	1	1	5	63
*Stagonospora* sp.	37	168	0	0	4	14	5	33	1	2	6	69
**Full predicted secretome**												
*A*. *alternata*	48	172	8	10	9	26	8	44	4	26	7	108
*P*. *sporulosum*	54	211	6	6	9	31	8	51	5	24	7	102
*Pyrenochaeta* sp.	51	181	8	11	7	34	8	52	6	22	6	89
*Stagonospora* sp.	50	183	8	9	9	24	8	50	4	11	7	104

The rich functional diversity of CAZymes extended to the predicted secretomes as well. The full predicted secretomes consistently included more families within each CAZyme class than the experimental secretomes, and the predicted secretomes contained increased numbers of proteins in sparsely populated families such as GT and polysaccharide lyases (PL) (Table 1).

### Secretome diversity among protein families

**Fig 2:**
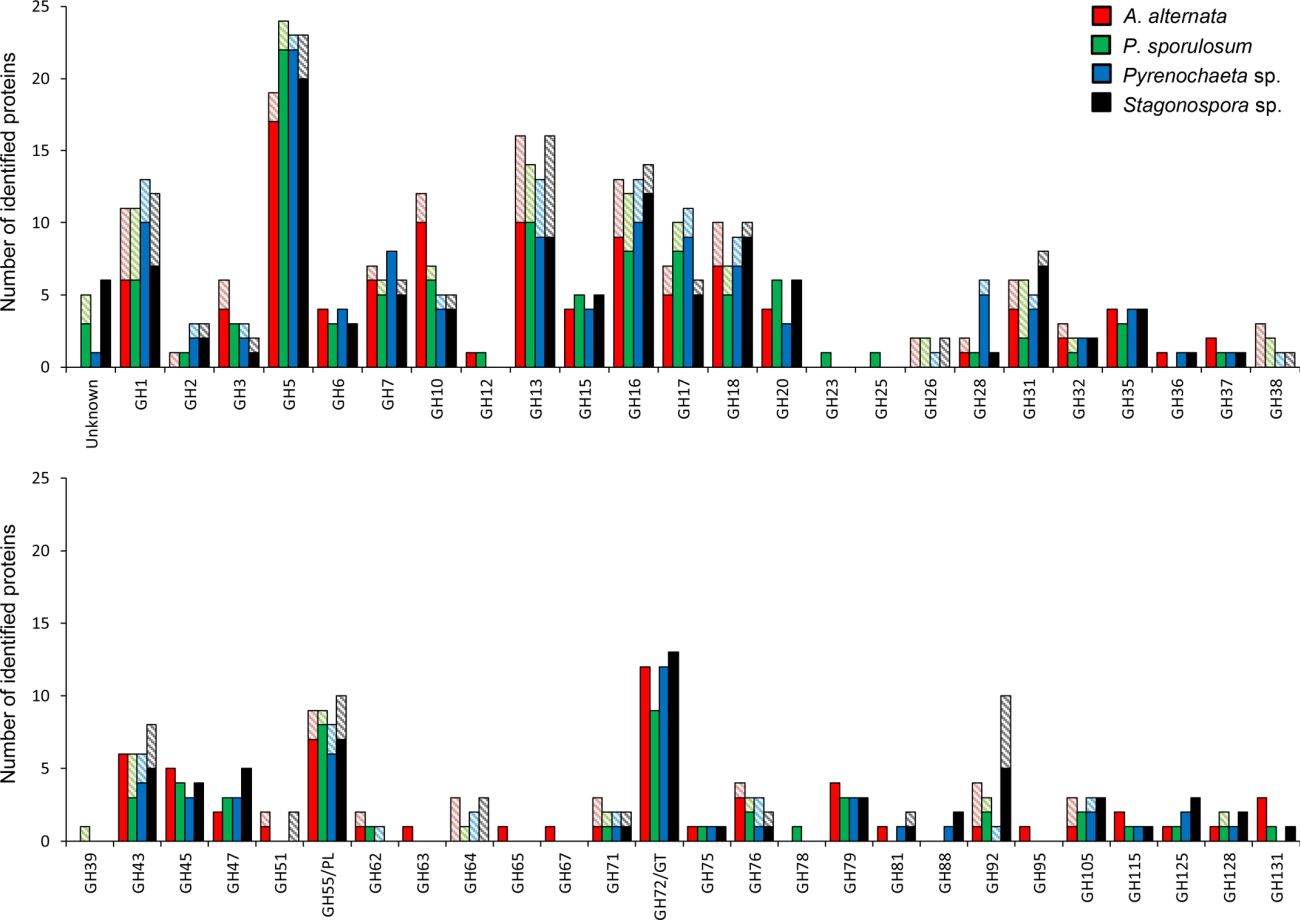
Distribution of proteins experimentally identified in secretomes of four Ascomycete fungi among protein families. Glycoside hydrolase families (both top and bottom panels). Solid bars: Portion of experimental secretome predicted to be secreted based on genome analysis. Shaded bars: Portion not predicted to be secreted. Proteins identified via LC-MS/MS over a 21-day study and classified according to the CAZy database. Abbreviations as in Fig 1.

**Fig 3:**
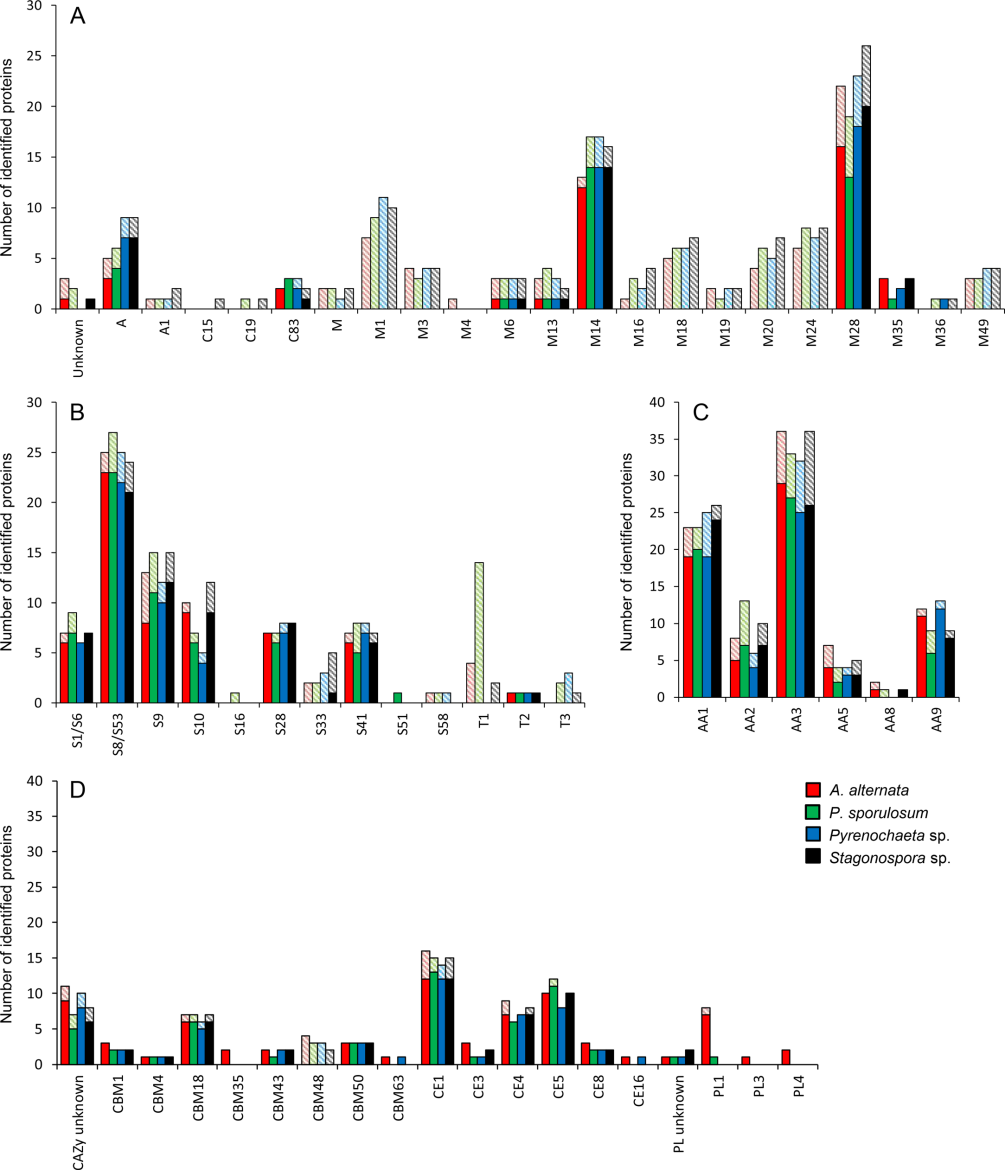
Distribution of proteins experimentally identified in secretomes of four Ascomycete fungi among protein families. (A, B) MEROPS peptidase families. (C) CAZy auxiliary activity families. (D) Other CAZyme families. Solid bars: Portion of experimental secretome predicted to be secreted based on genome analysis. Shaded bars: Portion not predicted to be secreted. Proteins identified via LC-MS/MS over a 21-day study and classified according to the CAZy and MEROPS databases. Abbreviations as in Fig 1.

Interestingly, when secretome functional diversity is examined at a deeper level, focusing on individual protein families within CAZy classes, the marked similarity across the four fungi persists (Figs 2 and 3). Furthermore, the rich diversity of identified CAZymes in each organism is more clearly illustrated. Within the GH class (Fig 2), the most frequently identified protein families included GH1 (cellulose-degrading β-glucosidases), GH5 (a diverse family of cellulases and hemicellulases), GH13 (a large family dominated by starch-degrading α-amylase), GH16-17 (diverse cellulases and hemicellulases), GH18 (chitinase), GH55/PL (β-1,3-glucanase), and GH72/GT (β-1,3-glucanosyltransglycosylase). The most well-represented class of GHs was GH5 with 19–24 proteins identified in each organism. Other CAZy families (Fig 3D) that were well-represented in the secretomes included CBM18 (carbohydrate-binding modules with demonstrated chitin-binding function), CE1 (mainly carboxylesterases and acetyl xylan esterases), CE4 (polysaccharide deacetylases), CE5 (cutinase), and CAZymes whose family designations were unknown.

While the GH class is primarily comprised of enzymes involved in the degradation of carbohydrates (e.g., cellulases, hemicellulases, starch), enzymes active in fungal cell wall degradation are also included. These cell wall-degrading enzymes include chitinases in the GH18 family, chitosanases in the GH75 family, chitin-binding proteins in various GH families, and β-1,3-glucanases in the GH55 and GH64 families [11]. Enzymes from each of these groups were identified in all 4 fungal secretomes and ranged from 13% to 15% (24–31 proteins) of the total number of identified proteins in the GH class in each secretome (Fig 2).

Among redox-active AAs (Fig 3C), the AA3 family (GMC oxidoreductases, predominantly with FAD cofactors) contained the highest number of identified proteins (an average of 34) in each fungus, and included choline dehydrogenases, cellobiose dehydrogenases, and alcohol oxidases. Notably, numerous cellobiose dehydrogenases were identified in all 4 organisms. The AA1 family (multicopper oxidases) was also well represented, with an average of 24 proteins per fungus; identified proteins in this family included ascorbate oxidase, bilirubin oxidase, coproporphyrinogen III oxidase, tyrosinase, and two proteins that mapped to traditional fungal laccases. Neither of the laccases were identified in the *Pyrenochaeta* sp. secretome, although this organism produced numerous other multicopper oxidases for which specific enzyme names were unavailable. The AA2 (class II peroxidase) family was only moderately represented in these Ascomycete secretomes and was comprised primarily of catalases. Lignin-degrading peroxidases typically found in Basidiomycetes were rare, with one ligninase and one heme peroxidase identified; at least one of these enzymes was identified in each of the 4 fungi. The AA5 (radical copper oxidase) family was even less well represented, consisting of one glyoxal oxidase, five galactose oxidases, and several unspecified radical copper oxidases; enzymes of each type were identified in all 4 fungi. Three iron reductases, two of which are annotated as having cellulose-binding capacity, in the AA8 family were identified, although none appeared in the *Pyrenochaeta* sp. secretome. Finally, numerous lytic polysaccharide monooxygenases in the AA9 family (formerly GH61) were identified in all 4 fungi.

Like the CAZy protein families, the functional diversity among MEROPS peptidase families was high in each organism and remained similar across the four fungi (Fig 3A and 3B). Peptidase families that were well populated in the secretomes included metallo- (M) and serine-type (S) peptidases such as M1 (aminopeptidase), M14 (carboxypeptidases), M28 (aminopeptidases and carboxypeptidases), S8/S53 (subtilisin/kexin/sedolisin, tripeptidyl peptidase I, and oryzin), S9 (dipeptidyl peptidase IV/V), and S10 (carboxypeptidases). While metallo- and serine-type peptidases were the most frequently observed in the secretomes, other identified peptidase classes included aspartic (A), cysteine (C), and threonine (T); no glutamic-type (G) or mixed (P) peptidases were identified.

Despite the striking similarity among the four organisms, a few exceptions were identified in which protein families were dominated by proteins from a single fungus. Approximately twice as many proteins were identified in the GH3 (cellulase and hemicellulase) and GH10 (endo-1,4-β-xylanase) families in the *A*. *alternata* secretome than in any of the other three fungi, and several GH families (GH 63, 65, 67, and 95) were identified only in *A*. *alternata* (Fig 2). Additionally, polysaccharide lyases (PLs), other than GH55/PL family proteins, were almost exclusively identified in the *A*. *alternata* secretome and consisted predominantly of PL1 family proteins (pectate lyases) (Fig 3D). Like that of *A*. *alternata*, the *P*. *sporulosum* secretome included several GH families identified exclusively in this organism’s secretome (GH23, 25, 39, and 78) (Fig 2). The *Pyrenochaeta* sp. secretome exhibited more than twice as many identified proteins in the GH28 (pectinase) family than that of any other organism, while the GH92 (α-mannosidase) family was dominated by *Stagonospora* sp. proteins (Fig 2). Finally, more than three times as many proteins were identified in the MEROPS T1 family (proteasome peptidases involved in intracellular protein turnover) in the *P*. *sporulosum* secretome than in that of any other fungus (Fig 3B).

Although “other” proteins were too numerous and varied to summarize concisely in distinct families, enzymes potentially involved in lignocellulose degradation were indeed observed. Particularly noteworthy were proteins potentially contributing to quinone redox cycling, including copper-containing amine oxidases with quinone-binding capability, which were identified in all 4 fungi (S1 Table).

Not all experimentally observed proteins were predicted to be secreted, and the proportion of proteins predicted to be secreted varied among functional classes and protein families (Figs 2 and 3). On average, 78%, 78%, and 86% of experimentally observed GHs, AAs, and other CAZymes, respectively, were predicted to be secreted, while only 56% of peptidases exhibited secretion signals. GH families such as GH35 (cellulose-degrading β-galactosidase) and GH72/GT exhibited a high proportion of proteins predicted to be secreted, while families such as GH1 and GH13 contained a larger proportion of proteins not predicted to be secreted (Fig 2). Four GH families (GH26, GH38, GH39, and GH64) were represented exclusively by proteins not predicted to be secreted. In contrast, nearly one-half of all peptidase families (17 out of 35) represented in the experimental secretomes did not contain any proteins predicted to be secreted (Fig 3A and 3B). Of these, families M1, M18 (aminopeptidases), M20 (carboxypeptidases and dipeptidases), and M24 (aminopeptidases) contained the highest number of identified proteins. Notably, no MEROPS family T1 peptidases (intracellular proteasome peptidases) were predicted to be secreted.

### Proteins unique to each fungus

**Fig 4:**
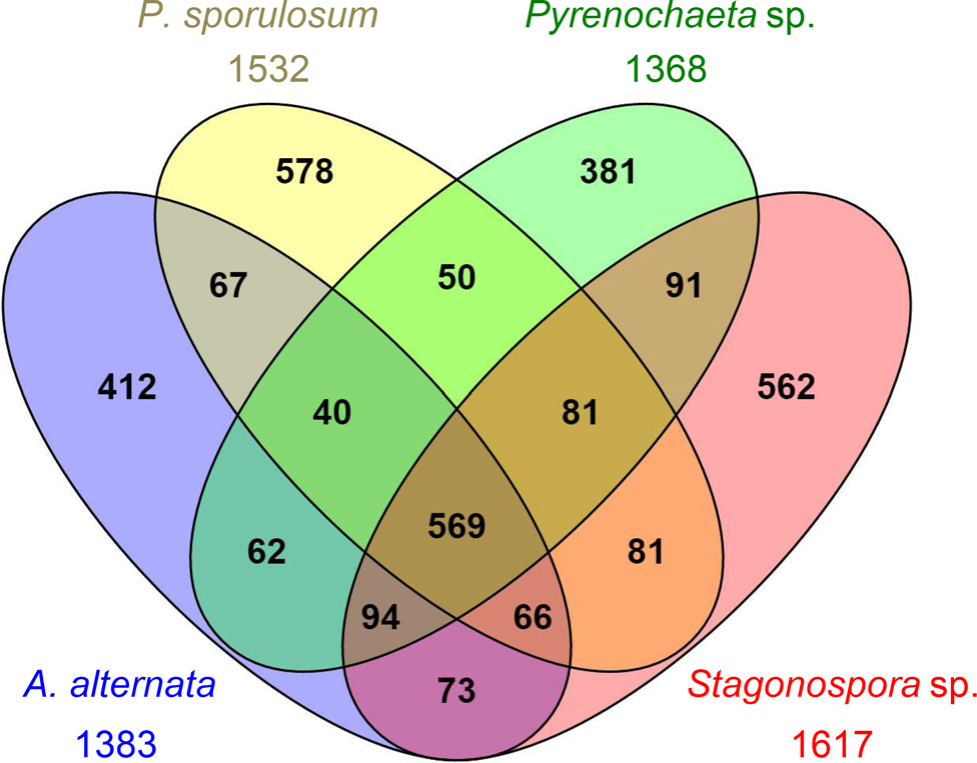
Venn diagram showing number of unique and shared proteins experimentally identified in Ascomycete fungi secretomes. Proteins identified via LC-MS/MS over a 21-day study. Total number of proteins identified for each fungus is indicated outside of diagram. Diagram generated with Venny 2.0 [*Oliveros*, *J*.*C*. *(2007–2015) Venny*. *An interactive tool for comparing lists with Venn’s diagrams. http://bioinfogp.cnb.csic.es/tools/venny/index.html*].

Notwithstanding the pronounced similarities in protein composition of the experimental secretomes among the four fungi, we identified comparable levels of unique and shared amino acid sequences among the organisms (Fig 4). A total of 569 identified proteins were shared among all four organisms, comprising 35% (in *Stagonospora* sp.) to 42% (in *Pyrenochaeta* sp.) of the total number of identified proteins in each fungus. Similarly, the number of proteins uniquely identified in each organism ranged from 28% (in *Pyrenochaeta* sp.; 381 proteins) to 38% (in *P*. *sporulosum*; 578 proteins) of the total for each fungus. The secretomes of *Pyrenochaeta* sp. and *Stagonospora* sp. exhibited the highest degree of similarity, with 835 shared sequences, while lower levels of shared sequences were observed between these two fungi and either *A*. *alternata* or *P*. *sporulosum*. Among pairs of fungi, the *P*. *sporulosum* and *Pyrenochaeta* sp. secretomes displayed the lowest level of similarity with 740 shared sequences.

Examination of unique and shared protein sequences among individual GH families yields further insight into the degree of interspecies similarity among the four experimentally observed fungal secretomes. While most GH families contained proteins that were identified in more than one fungus, the extent to which unique versions of these enzymes were identified in individual fungi varied among the families (Table 2). For instance, while GH20 and GH35 families comprised primarily (≥80%) shared proteins, GH3 and GH92 families contained predominantly species-specific sequences (with only 20% of proteins shared by more than one fungus). Moreover, it is noteworthy that no GH families containing more than 3 identified proteins were represented exclusively by shared sequences; thus, species-specific versions of functionally similar enzymes (i.e., potential isozymes) were an inherent characteristic of these fungal secretomes. The example shown here for GH families was chosen for its rich diversity, but the patterns identified herein were representative of other CAZy and MEROPS protein families (data not shown).

**Table 2:** Number of shared and unique experimentally identified proteins in glycoside hydrolase families among the four fungal secretomes.

GH Family	Percent shared	Total	Shared		Unique to one fungus			
			4 fungi	2–3 fungi	*A*. *alt*.	*P*. *spor*.	*Pyreno*.	*Stago*.
**GH26**	100%	2	1	1	0	0	0	0
**GH62**	100%	2	0	2	0	0	0	0
**GH64**	100%	3	1	2	0	0	0	0
**GH75**	100%	1	1	0	0	0	0	0
**GH20**	86%	7	1	5	0	1	0	0
**GH1**	81%	16	8	5	0	0	2	1
**GH7**	80%	10	3	5	0	0	2	0
**GH35**	80%	5	2	2	1	0	0	0
**GH47**	80%	5	1	3	0	0	0	1
**GH79**	75%	4	3	0	1	0	0	0
**GH15**	67%	6	4	0	0	1	0	1
**GH38**	67%	3	1	1	1	0	0	0
**GH71**	67%	3	2	0	1	0	0	0
**GH72/GT**	67%	18	7	5	1	1	1	3
**GH17**	64%	14	5	4	1	2	2	0
**GH18**	63%	16	3	7	2	1	1	2
**GH55/PL**	62%	13	7	1	1	2	0	2
**GH105**	60%	5	1	2	1	0	0	1
**GH5**	54%	41	9	13	2	6	6	5
**GH6**	50%	6	2	1	1	1	1	0
**GH36**	50%	2	0	1	0	0	0	1
**GH37**	50%	2	1	0	1	0	0	0
**GH88**	50%	2	0	1	0	0	0	1
**GH115**	50%	2	1	0	1	0	0	0
**GH131**	48%	27	9	4	6	3	2	3
**GH43**	47%	15	0	7	2	3	1	2
**GH16**	44%	25	7	4	4	3	3	4
**GH2**	40%	5	0	2	1	0	1	1
**GH32**	40%	5	1	1	1	1	0	1
**GH45**	38%	8	2	1	2	2	0	1
**GH105**	38%	16	2	4	6	2	1	1
**GH31**	33%	15	2	3	1	4	2	3
**GH81**	33%	3	0	1	0	0	1	1
**GH128**	33%	3	1	0	0	1	0	1
**Unknown**	33%	9	0	3	0	3	0	3
**GH28**	25%	8	0	2	1	0	4	1
**GH125**	25%	4	1	0	0	0	1	2
**GH3**	20%	10	1	1	5	1	1	1
**GH92**	20%	15	0	3	2	2	0	8
**GH76**	11%	9	1	0	3	2	2	1
**GH12**	0%	2	0	0	1	1	0	0
**GH23**	0%	1	0	0	0	1	0	0
**GH25**	0%	1	0	0	0	1	0	0
**GH39**	0%	1	0	0	0	1	0	0
**GH51**	0%	4	0	0	2	0	0	2
**GH63**	0%	1	0	0	1	0	0	0
**GH65**	0%	1	0	0	1	0	0	0
**GH67**	0%	1	0	0	1	0	0	0
**GH78**	0%	1	0	0	0	1	0	0
**GH95**	0%	1	0	0	1	0	0	0
**GH131**	0%	5	0	0	3	1	0	1
**Total**	—	384	91	97	59	48	34	55

*A*. *alt*. = *A*. *alternata*; *P*. *spor*. = *P*. *sporulosum*; *Pyreno*. = *Pyrenochaeta* sp.; *Stago*. = *Stagonospora* sp. Percent shared = total number of proteins shared between 2–4 fungi divided by total number of proteins identified.

**Fig 5:**
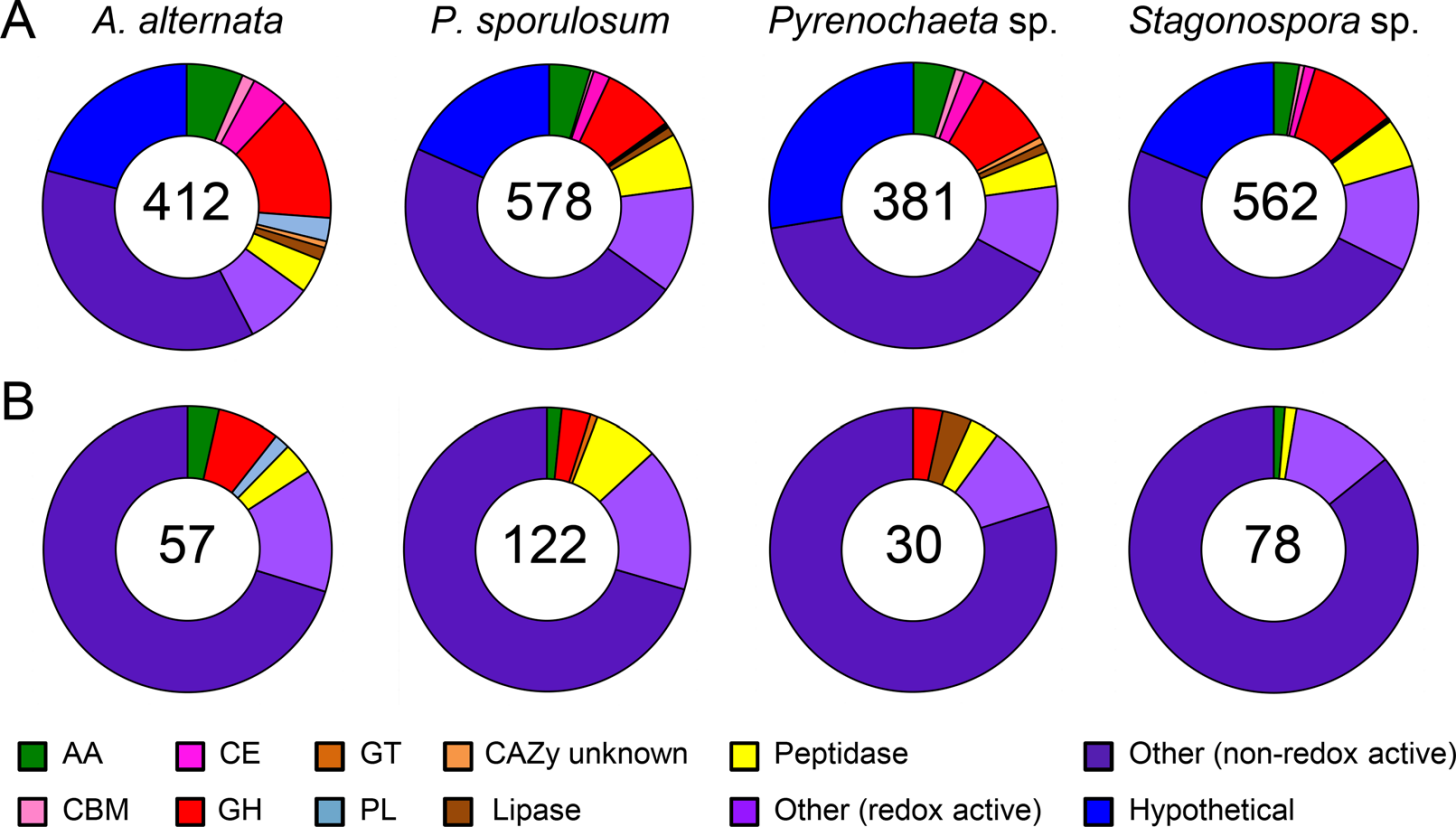
Distribution of unique proteins experimentally identified in secretomes of four Ascomycete fungi. (A) Proteins unique to each fungus based on amino acid sequence (as determined by JGI protein ID). (B) Proteins unique to each fungus based on predicted function (evaluated manually). Proteins identified via LC-MS/MS over a 21-day study. Total number of unique proteins identified for each fungus is indicated in center of circles. Abbreviations as in Fig 1.

Among proteins experimentally identified in the secretomes, proteins unique to each fungus spanned the full range of broad CAZy and MEROPS functional groups that were identified in the full secretomes (Fig 5A). However, the proportion of CAZymes was considerably lower among unique proteins (ranging from 15% in *Stagonospora* sp. to 18% in *Pyrenochaeta* sp.) than in the whole secretomes, except in *A*. *alternata* where CAZymes comprised 30% of unique sequences. This difference was primarily attributed to the large number of unique GHs (59 proteins) identified in the *A*. *alternata* secretome and correlates with observations of unique GH families in this organism (Fig 2), as discussed above. Few unique peptidases (4–6% of the total number of identified proteins) were identified in the secretomes of each fungus, while the majority of unique sequences consisted of “other” (44–61%) and hypothetical (18–28%) proteins. The “other” proteins exhibited a large range of functional diversity and included many intracellular proteins that may have been released via lysis during growth or sample processing. Complete lists of proteins uniquely identified in the experimental secretome of each fungus is presented in S6–S9 Tables.

Manually examining the predicted function of the experimentally identified proteins unique to each fungus revealed that only a small subset (ranging from 30 proteins in *Pyrenochaeta* to 122 proteins in *P*. *sporulosum*) of unique protein sequences were truly functionally unique to each organism (Fig 5B), thereby reinforcing the levels of interspecies similarity in secretome functional diversity as discussed above. The proportion of functionally unique CAZymes was very low (ranging from 12% in *A*. *alternata* to only 1% in *Stagonospora* sp.), while the vast majority of functionally unique enzymes identified in the secretomes were “other” proteins. (Hypothetical proteins were excluded from this analysis since their function is unknown.) In agreement with earlier observations, the *A*. *alternata* secretome exhibited the highest number of functionally unique identified CAZymes (7 proteins), including 4 GHs (1 each in GH63, GH65, GH67, and GH95 families) and 2 AAs (a vanadium chloroperoxidase in the AA2 family and a cellulose binding protein in the AA9 family) (S6 Table). The *P*. *sporulosum* secretome contained the largest number of functionally unique peptidases (9 proteins, 6 of which were MEROPS T1 proteasome peptidases) (S7 Table), while only two non-“other” functionally unique proteins were identified in the *Stagonospora* sp. secretome (a peroxidase in the AA2 family and a MEROPS C15 peptidase) (S9 Table). Functionally unique proteins identified in the *Pyrenochaeta* sp. secretome included a pectin-degrading rhamnogalacturonase in the GH28 family, an aspartic peptidase (candidapepsin), an unspecified lipase, and numerous “other” proteins (S8 Table). Notably, several “other” proteins potentially involved in quinone redox cycling were functionally unique to the *P*. *sporulosum* secretome, including a hydroxyquinol 1,2-dioxygenase, two quinone oxidoreductases, and a quinate permease (S7 Table).

Results of the OrthoMCL analysis revealed that few of the experimentally observed unique and functionally unique proteins identified in each secretome were actually unique to each genome (Table 3). Of experimentally observed unique proteins, only 3–9% were unique to the genome of each species, and only 1–4% were both unique to the genome and predicted to be secreted. Even fewer experimentally observed functionally unique proteins were unique to each genome, with no more than 9 proteins with these characteristics identified in each fungus, and no more than 3 predicted to be secreted.

**Table 3:** Genome-based evaluation of unique proteins experimentally identified in secretomes of four Ascomycete fungi.

Proteins identified in experimental secretomes:	Fungal species			
	*A*.*alternata*	*P*. *sporulosum*	*Pyrenochaeta* sp.	*Stagonospora* sp.
**Protein sequences unique to each species**	* *	* *		
Total	412	578	381	562
Predicted to be secreted	169	146	136	151
Unique to species genome	35	44	19	21
Unique to species genome and predicted to be secreted	16	23	9	7
**Proteins functionally unique to each species**				
Total	57	122	30	78
Predicted to be secreted	17	9	5	10
Unique to species genome	9	4	1	2
Unique to species genome and predicted to be secreted	3	2	0	1

Proteins identified via LC-MS/MS over a 21-day study. Genome analysis based on OrthoMCL (see text for details).

**Fig 6:**
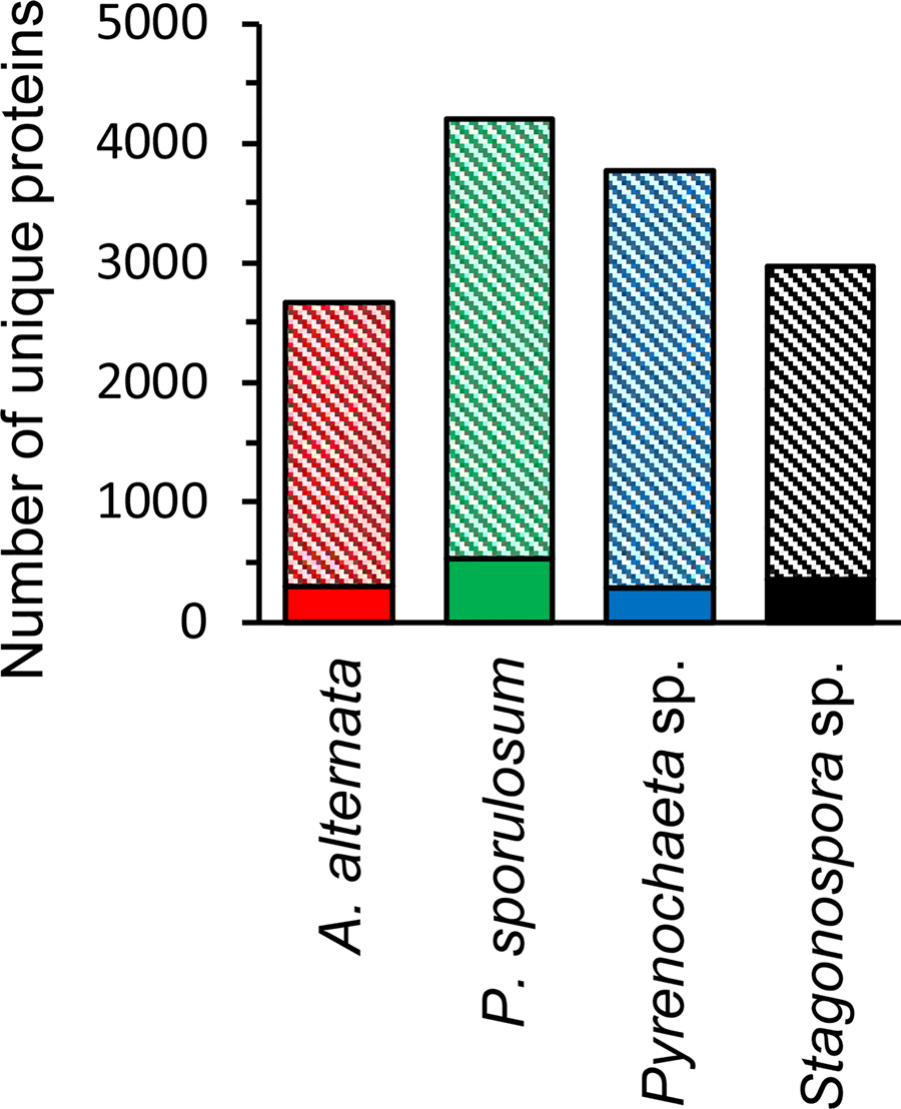
Proteins unique to each genome among four Ascomycete fungi. Genome analysis based on OrthoMCL (see text for details). Data are solely based on genomes and do not include experimental data. Solid bars: unique proteins predicted to be secreted. Shaded bars: unique proteins not predicted to be secreted. Colors as in Figs 2 and 3. Total number of predicted proteins in each genome (including unique and non-unique): 13,469 (*A*. *alternata*); 14,745 (*P*. *sporulosum*); 14,995 (*Pyrenochaeta* sp.); 14,430 (*Stagonospora* sp.).

Finally, OrthoMCL analysis of the entire genomes of each of the four fungi indicated that 20–28% of the predicted proteins in each genome were unique to each species, ranging from 2,668 proteins in the *A*. *alternata* genome (out of a total of 13,469 proteins) to 4,201 proteins in the *P*. *sporulosum* genome (out of a total of 14,745) (Fig 6). Of these, 8–13% were predicted to be secreted, ranging from 290 proteins in *Pyrenochaeta* sp. to 530 in *P*. *sporulosum*. These genomically unique proteins that were predicted to be secreted represented only 2–4% of the proteins in the genomes.

## Discussion

### Secretome size

The secretomes we characterized in this study are large and are generally in agreement with published genome-based estimates. In a computational study of the impact of phylogeny and fungal lifestyle on secretome size, Krijger et al. [46] predicted a mean of approximately 900 proteins for *Pezizomycotina*, the Ascymycota sub-phylum to which the four fungi in this study belong. This estimate varied by lifestyle, ranging from a mean of approximately 600 proteins for saprotrophs to approximately 1,200 proteins for plant pathogens. Additionally, they estimated a secretome size of between approximately 600 and 1,500 proteins in fungi with genome sizes between 30 and 40 Mb. With secretome sizes of approximately 1,300–1,600 proteins (for both experimental and genome-based predicted secretomes) (Fig 1) and genome sizes of 33 to 39 Mb (JGI Mycocosm), the Ascomycetes we studied, which can act as both saprotrophs and plant pathogens, sit at the high end of these estimates. As it has been reported that secretome sizes predicted by gene sequences may substantially underestimate the total number of secreted proteins [57], the large secretomes characterized in this study may indeed be representative of fungi with similar taxonomy and lifestyle. A conservative estimate (518–576 proteins) of the experimental secretome size that excludes “other” and hypothetical proteins, whose functions are unknown and/or potentially intracellular, still places these fungi within or near the genome-based estimates for sub-phylum and genome size and is comparable to the estimated secretome size for saprotrophs. Likewise, the portion of the experimentally observed secretomes that is predicted to be secreted (approximately 700 proteins per species), falls within the range of the Krijger et al. [46] estimates.

Comprehensive experimental secretome studies of other filamentous fungi have generally reported less than 350 proteins identified in the secretomes of filamentous Ascomycetes [6, 9, 11] and Basidiomycetes [8, 14] based on protein harvests and LC/MS/MS analysis, including iTRAQ proteomic studies [5, 13, 58], although these numbers are increasing over time concomitant with technological advancements. Nonetheless, even when we consider only those experimentally observed proteins that are predicted to be secreted, we identified substantially larger secretomes that consisted of 634–698 proteins per species. As we used standard sample preparation and protein digestion techniques, the large secretome sizes may be associated with the ability of the fungi to act as plant pathogens, the efficacy with which the iTRAQ-based proteomic workflow employed herein can identify low-abundance proteins, or a combination of these. Regardless, the large secretomes identified herein shed new light on the rich functional diversity of extracellular proteins of these organisms.

Interestingly, the large genome-based predicted secretomes presented in this study still appear to underestimate the full protein diversity of these organisms. CAZymes such as specific carbohydrate-degrading GHs and non-specific carbon-oxidizing AAs are generally thought to be extracellular enzymes, secreted specifically for uptake of extracellular substrates. Here, however, we report that only about 80% of the proteins that we experimentally identified in these classes were predicted to be secreted. Furthermore, we experimentally observed nearly twice as many peptidases as those predicted to be secreted, including many well-represented families, suggesting that the genome-based estimates may underestimate peptidase secretion (although the extent to which this may be occurring is unknown).

Finally, although we evaluated the experimentally observed proteins and genome-based predicted proteins for the presence of a secretion signal peptide and transmembrane domains, we did not evaluate the potential for non-classical secretion pathways due to high error rates in existing online tools used for this purpose. Thus, non-classical secretion pathways may indeed be present in these fungi and could add to the underestimation of protein diversity by the genome-based predicted secretomes.

### Functional diversity of the secretome

The striking similarity in functional diversity of the secretomes among the four Ascomycetes in this study, in addition to the differences with respect to earlier reports, can likely be attributed to substrate and growth conditions. It is well established that the suite of extracellular enzymes secreted by fungi is highly dependent on substrate composition, and that functional diversity increases when the organisms are presented with more complex and recalcitrant material (reviewed in [59]; demonstrated in an early secretome study in by Phalip et al. [12]). Here, we cultured four fungi under identical growth conditions using the same, easily digestible substrate, with acetate and yeast extract (roughly 70% protein and 15% carbohydrates) [60] as carbon sources, resulting in similar proportions of identified GHs and peptidases in the experimentally observed secretomes (Fig 1). In contrast, Liu et al. [5] observed substantially more cellulases and hemicellulases than peptidases secreted by *Aspergillus fumigatus* Z5 (in the same sub-phylum as the fungi in this study) growing on cellulosic substrates, with few identified oxidoreductases, and a marked increase in oxidoreductases when the carbon source was switched to glucose. Similarly, Hori et al. [14] reported a large ratio of GHs to peptidases secreted by the white-rot Basidiomycete *Ceriporiopsis subvermispora* during growth on aspen wood, with roughly equal proportions of peptidases and redox-active AAs.

Notwithstanding the phylogenetic differences between these organisms and the Mn(II)-oxidizing fungi in this study, these data suggest that the proportion of GHs would similarly increase when growing these fungi on plant-derived substrates, as more cellulases and hemicellulases would likely be required to utilize this more recalcitrant carbon source. Thus, the functional diversity of the secretomes of these fungi may be even greater than our experimental data suggest. As it is likely that the genome-based predicted secretomes still underestimate the true functional capacity of these fungi, as discussed above, even the higher estimates of CAZymes in the full predicted secretomes may still not be representative of the true carbon-degradation potential of these organisms.

It is probable that the relative proportion of AAs identified in fungal secretomes is dependent on both substrate and phylogeny, as the larger proportion of AAs reported by Hori et al. was primarily driven by an abundance of peroxidases in the AA2 family, characteristic of Basidiomycete lignin degraders [1, 2].

#### Carbohydrate-degrading enzymes

The wide variety of extracellular hydrolytic enzymes identified in the secretomes of these Ascomycetes is noteworthy, particularly in light of the relatively simple, non-cellulosic substrate provided. Between 40 and 45 families of glycoside hydrolases and up to 214 individual proteins in this class were experimentally identified in each organism, with more diversity predicted based on the genomes (Table 1). For comparison, other studies that classified secretome data in a similar manner reported 11 [11], 20 [12], or 39 [9] GH families identified in other filamentous Ascomycete secretomes and 31 [14] and 35 [15] GH families identified in those of white-rot Basidiomycetes; all of these studies except Nitsche et al. [11] (11 GH families) represented growth on plant-based substrate. Notably, a genome-based estimate of 42 GH families in the predicted secretome of *Stagonospora nodorum* [46] correlates well with our laboratory-based identification of 41 GH families in *Stagonospora sp*. SRC1lsM3a.

The diverse range and large quantities of carbohydrate-degrading enzymes identified in the secretomes of these Ascomycetes suggests that their carbon oxidative capacity is robust. The secretomes contained identified proteins spanning a large range of cellulases (e.g., glucanases, glucosidases, galactosidases), hemicellulases (e.g., xylanases, mannases), and starch-degrading enzymes (α-amylase), rather than being dominated by a single type of hydrolytic enzyme (Fig 2). These data suggest a synergistic approach to carbon degradation among diverse extracellular enzymes in these organisms. The profiles of identified GHs and other CAZymes presented in this study, particularly frequently observed families such as GH1, 5, 16, and 17, may be used as a baseline for future investigations of the mechanisms underlying the cellulose degrading capacity of these organisms. The presence of cell wall-degrading enzymes in each of the fungal secretomes, in addition to carbohydrate-degrading enzymes, suggests that these organisms undergo biomass recycling concomitant with primary substrate utilization.

#### Auxiliary activities and potential Fenton-based lignocellulose oxidation

The profiles of redox-active AAs identified in the secretomes of these Mn(II)-oxidizing fungi (Fig 3C) suggest that the cellulose degradation capacity of these organisms may involve both direct carbohydrate breakdown via glycoside hydrolases as well as indirect carbon oxidation via Fenton-based hydroxyl radical formation. The combination of H_2_O_2_-generating enzymes (e.g., GMC oxidoreductases [61] and radical copper oxidases [62, 63]), enzymes involved in hydroquinone redox cycling (e.g., cellobiose dehydrogenase and [64] tyrosinase [65]), and Fe(III) reductases could supply the necessary reagents for Fenton chemistry in the presence of ferrous iron. While it has been demonstrated that the production of high levels of oxalate can form Fe(III)-oxalate chelates that are poorly reduced by hydroquinones [66], future metabolomic characterization of the secretomes of these organisms would be necessary to evaluate the potential inhibitory effect of secreted oxalate on any Fenton-based carbon degradation mechanisms in these fungi.

Similarly, the production of extracellular antioxidant enzymes by the fungi could reduce or inhibit ROS-based carbon degradation mechanisms. Antioxidant enzymes were identified in all 4 experimental secretomes (ranging from 21 enzymes in the *A*. *alternata* and *Pyrenochaeta* sp. secretomes to 32 enzymes in the *Stagonospora* sp. secretome) and included superoxide dismutases, catalases and peroxidases in the AA2 family, peroxiredoxins, glutathione reductase, and other enzymes involved in the glutathione antioxidant system (S1 Table). Again, future metabolomic and chemical characterization of the secretomes of these organisms would be necessary to evaluate any potential inhibitory effects on carbon degradation mechanisms.

Although the lignin-degrading capacity of these fungal strains has not been evaluated and remains unknown, a non-specific, Fenton-based carbon oxidation mechanism could potentially attack phenolic as well as cellulosic substrates. This possibility is particularly intriguing in *P*. *sporulosum*, where we observed several enzymes involved in quinone redox cycling that were not present in the other 3 fungi, including hydroxyquionol 1,2-dioxygenase which is thought to play a role in aromatic metabolism of lignin in *Phanerochaete chyrosporisum* [67]. The secretomes of these Ascomycetes lacked traditional lignin-degrading enzymes such as manganese and versatile peroxidases and had a substantially smaller proportion of AA2 enzymes than in published Basidiomycete secretomes [14] and transcriptomes [15]. However, the diversity and high number of other identified AAs, including two laccases, which can degrade diverse phenolic compounds directly as a substrate (reviewed in [68]) and indirectly through Mn(II) oxidation and ROS production [22, 23], is promising and warrants further investigation into the lignocellulolytic degradation potential of these organisms. The possibility of a Fenton-based lignocellulose degradation mechanism in these Ascomycetes is intriguing given that environmental lignin degradation is thought to be carried out primarily by Basidiomycete fungi.

#### Peptidases

While we identified a wide variety of peptidases in the secretomes of these Ascomycetes (Fig 3A and 3B), the relative functional diversity of these organisms compared to other filamentous fungi is difficult to assess since few secretome studies have published family-level peptidase classifications.

The most frequently identified peptidases in this study, S8/S53 family peptidases, represent a large and diverse group of serine-type proteases whose biological function does not appear to be well characterized in fungi. Family S8 proteins (subtilisins) have been identified in nearly all domains of life and are thought to be of ancient origin [69], and family S53 proteins (serine carboxypeptidases known as sedolisins) are known for one member, CLN2, involved in a human neurodegenerative disorder [70, 71]. Our identification of numerous family M14 zinc carboxypeptidases agrees well with observations by Krijger et al. [46] that this domain is expanded in a variety of plant pathogenic *Pezizomycotina*; these peptidases typically degrade extracellular matrix proteins and are thought to play a role in cell defense against attack by a plant host. Overall, the diverse complement of peptidases identified in these Ascomycetes suggests that many structurally diverse proteins can be degraded by these organisms. In this study, these peptidases may have contributed to consumption of the proteinaceous yeast extract substrate during growth and aided in fungal cell wall remodeling and nutrient recycling as the cultures aged and substrate likely became scarce [11].

The large proportion of peptidases that we experimentally observed in the secretomes but were not predicted to be secreted is noteworthy. This may indicate that the genome-based predicted secretomes underestimate the full complement of secreted peptidases in these species, as discussed above. For instance, peptidase families such as M14 and S8/S53 in which most, but not all, proteins are predicted to be secreted may indicate underestimation. Alternatively, these peptidases could represent intracellular proteins that were released into the spent medium via lysis. Families in which no proteins were predicted to be secreted, such as T1 proteasome peptidases involved in intracellular protein turnover, may reflect this phenomenon. It is intriguing, however, that the experimentally observed suite of non-secreted peptidases was so much greater than that of other carbon-degrading enzyme functional classes. This may indicate that the complement of intracellular proteins in these fungi that would be released upon lysis contains substantially more peptidases than carbon-degrading enzymes, which would not be surprising given that CAZymes are typically extracellular enzymes. Future investigation of the function of the experimentally observed peptidases in Ascomycete fungi may shed light on our results.

#### “Other” proteins

The identification of intracellular proteins in the experimentally observed secretomes is further supported by the decrease in “other” (non-CAZy or MEROPS) proteins in the predicted secretomes. A total of 649–850 “other” proteins were identified in the experimental secretomes, depending on the species, but only 184–210 of these were predicted to be secreted (Fig 1), suggesting that many were indeed of intracellular origin. For example, isocitrate lyase, an intracellular protein involved in energy generation, was identified in the experimental secretomes of each species but not the predicted secretomes (S1–S5 Tables). The predicted secretomes of each species still contain over 30% “other” proteins (compared to roughly 50% in the experimental secretomes), including proteins such as copper-containing amine oxidases that could contribute to quinone redox cycling.

#### Hypothetical proteins and proteins with limited functional information

The role of hypothetical proteins in the fungal secretomes remains ambiguous due to either the lack of functional information assigned to these proteins or the lack of a protein match with an acceptably low E value in the NCBI and UniProt databases. Hypothetical proteins comprise a non-negligible proportion (up to 14%) of identified proteins in the experimental secretomes and include up to 194 individual proteins (Fig 1). As such, at least some of these proteins likely play important roles in extracellular carbon transformations.

Interestingly, the predicted secretomes contain nearly three times as many hypothetical proteins (up to 580 per species) than the experimentally observed secretomes (Fig 1). It is possible that the genes that encode these proteins are rarely expressed under laboratory conditions and thus have received little or no characterization in previous studies. As secretome plasticity is dependent upon carbon source and growth conditions, these hypothetical proteins may represent uncharacterized functional diversity in these species.

Another challenge in working with large proteomic datasets, particularly for environmental isolates of fungal species for which relatively little proteomic information is available for closely-related organisms, is the incompleteness of functional information in existing fungal databases, even for non-hypothetical proteins. For instance, many of the proteins we identified in this study were mapped to proteins for which only family-level annotations were available (e.g, “glycoside hydrolase family 5” or “M18 metallopeptidase”), rather than a specific enzyme name, thus limiting our understanding of which enzymes are present and which reactions they may be catalyzing in the secretome.

Nevertheless, it is noteworthy that we were able to assign functional information to over 85% of peptides generated by these environmental isolates (i.e., non-model organisms) for which a protein match could be found in the sequenced genomes. This is particularly encouraging in light of the fact that species-level determination of two of our isolates, *Stagonospora* sp. and *Pyrenochaeta* sp., remains elusive. As research on fungal genomics and proteomics is rapidly increasing, we look forward to delving more deeply into datasets such as these as more information becomes available.

### Species-specific secretome characteristics

The Mn(II)-oxidizing Ascomycetes in this study produce a rich yet functionally similar suite of extracellular enzymes under the evaluated growth conditions, with species-specific differences arising from unique amino acid sequences rather than overall protein function. While our data indicate that up to 38% of the identified proteins in the experimental secretomes represent species-specific sequences (Fig 4) that span the full range of CAZy and MEROPS functional groups (Fig 5A), very few of these proteins confer unique functionality to the experimentally observed secretomes (Fig 5B). Of those that do, most were characterized as “other” proteins, many of which were likely of intracellular origin. Moreover, only a handful of experimentally observed unique proteins were actually unique to each fungal genome or predicted secretome (Table 3). Overall, the identification of few functionally unique, extracellular, carbon-degrading enzymes suggests that these hydrolytic and oxidative enzymes are well represented among all four fungi and that the organisms possess a similar carbon-degrading capacity under the evaluated growth conditions. The fact that less than 4% of predicted proteins in the genomes of these fungi were predicted to be both genomically unique and secreted (Fig 6) underscores the similarities in functional capacity of these species.

Of the few exceptions to this interspecies functional similarity that we identified at the protein family level (Figs 2 and 3), many may be dampened by functional redundancy within the secretomes. For example, while the GH63 family (α-glucosidases and α-mannonsidases) was identified exclusively in the *A*. *alternata* secretome, α-glucosidases in the GH31 family were identified in all four organisms, as were proteins in families GH38, GH47, GH76, and GH92, all of which contain α-mannonsidases. Notably, the predominance of GH92 family α-mannonsidases in the *Stagonorpora* sp. secretome is similarly offset by the presence of these functionally related families. The acid trehalase in the GH65 family and the α-glucoronidase in the GH67 family, both identified only in the *A*. *alternata* secretome, may share functionality with trehalases in the GH37 family and proteins in the GH115 family, respectively, both of which were identified in all four fungi. The GH23 and GH25 families, uniquely identified in the *P*. *sporulosum* secretome, are described as lysozymes, peptidoglycan lyases, and chitinases in the CAZy database; these families share similar functionality with GH18 family proteins, which were identified in the secretomes of all four organisms. Thus, even proteins identified as functionally unique based on GH family appear to be complemented by similar enzymes in other families, underscoring the levels of interspecies functional similarity among organisms. Future unambiguous identification of all identified proteins by enzyme name as opposed to family, which could be possible as fungal proteomic research progresses, would aid in clarifying these interspecies comparisons.

Exceptions to these observations of functional redundancy include an *o*-mannosyl-transferase in the GH39 family and an α-rhamnosidase in the GH78 family, both exclusively identified in the *P*. *sporulosum* secretome. However, only one protein of each type was identified, thus calling into question the significance of these unique proteins. Multiple MEROPS T1 proteasome peptidases were also uniquely identified in the *P*. *sporulosum* secretome. This finding may indicate increased intracellular protein recycling by *P*. *sporulosum* relative to the other three species.

Our finding that the four fungi in this study produce species-specific versions of functionally similar enzymes (Table 2) likely stems from the phylogenetic ancestry of the organisms. It has been previously demonstrated that phylogeny strongly influences fungal secretome composition, in addition to lifestyle adaptation (e.g., saprotroph vs. plant pathogen) and environmental conditions [46]. Here we demonstrate that the level of interspecies sequence similarity among secreted proteins is fairly consistent, ranging from 740 to 835 shared sequences (Fig 4), across the four fungi, all of which belong to the class *Dothideomycetes* and order *Pleosporales*. Taxonomic classifications within the *Pleosporales* order continue to undergo revision due to the insufficient resolution of 18S rDNA-based phylogenetic relationships [36]. As all four organisms are fairly closely related and exhibit comparable levels of sequence overlap, more detailed phylogenetic distinctions may not provide further insight into interspecies secretome similarity and may not be warranted given the uncertainties of phylogenetic relationships within the *Pleosporales* order.

## Conclusions

Here we have presented a first look at the protein composition of the secretomes of four filamentous Ascomycete fungi that are ubiquitous in soils and have the ability to degrade cellulose and generate strong oxidants such as Mn(III) and Mn(IV) oxides, which could contribute to lignin degradation. We have identified a rich yet functionally similar suite of extracellular hydrolytic and oxidative enzymes among the organisms growing on a complex medium, with species-specific differences in secretome composition arising from unique amino acid sequences rather than overall protein function. Furthermore, the identification of a diverse range of cellulases and hemicellulases, in combination with redox-active accessory enzymes that support ROS production and quinone redox cycling, suggests that the cellulose-degrading capacity of these organisms may involve both direct enzymatic carbohydrate breakdown as well as indirect carbon oxidation via Fenton-based hydroxyl radical formation. Thus, these Ascomycetes may contribute to lignocellulose degradation by wood-rot Basidiomycete fungi in the environment. Future investigations of these organisms on lignocellulosic substrate will further enhance our understanding of their role in environmental carbon turnover.

## Supporting Information

S1 Table All proteins experimentally identified in secretomes of *A*. *alternata*, *P*. *sporulosum*, *Pyrenochaeta* sp., and *Stagonospora* sp. (XLSX)

S2 Table Full genome-based predicted secretome of *A*. *alternata* (1352 proteins). (XLSX)

S3 Table Full genome-based predicted secretome of *P*. *sporulosum* (1604 proteins). (XLSX)

S4 Table Full genome-based predicted secretome of *Pyrenochaeta* sp. (1404 proteins). (XLSX)

S5 Table Full genome-based predicted secretome of *Stagonospora* sp. (1527 proteins). (XLSX)

S6 Table Proteins unique to *A*. *alternata* experimental secretome (412 proteins). (XLSX)

S7 Table Proteins unique to *P*. *sporulosum* experimental secretome (578 proteins). (XLSX)

S8 Table Proteins unique to *Pyrenochaeta* sp. experimental secretome (381 proteins). (XLSX)

S9 Table Proteins unique to *Stagonospora* sp. experimental secretome (562 proteins). (XLSX)
